# AMPD1 and MTHFR genes are not associated with calcium levels in rheumatoid arthritis patients with methotrexate therapy in Indonesia

**DOI:** 10.1038/s41598-024-69604-z

**Published:** 2025-01-10

**Authors:** Dika P. Destiani, Sumartini Dewi, Syed A. S. Sulaiman, Sofa D. Alfian, Melisa I. Barliana, Rizky Abdulah

**Affiliations:** 1 Department of Pharmacology and Clinical Pharmacy, Faculty of Pharmacy, Universitas Padjadjaran, Jatinangor, Indonesia; 2 Center of Excellence for Pharmaceutical Care Innovation, Jatinangor, Indonesia; 3 Division of Rheumatology, Department of Internal Medicine, Hasan Sadikin General Hospital, Bandung, Indonesia; 4 Imunology Study Centre, Faculty of Medicine, Universitas Padjadjaran, Bandung, Indonesia; 5 Department of Clinical Pharmacy, School of Pharmaceutical Sciences, Universiti Sains Malaysia, Penang, Malaysia; 6 Department of Biological Pharmacy, Faculty of Pharmacy, Universitas Padjadjaran, Jatinangor, Indonesia; 7 Center for Health Technology Assessment, Universitas Padjadjaran, Jatinangor, Indonesia

**Keywords:** Rheumatoid arthritis, Methotrexate, Serum calcium levels, Gene polymorphisms, Biotechnology, Genetics, Rheumatology

## Abstract

Rheumatoid Arthritis (RA) is a chronic and progressive autoimmune disease that affects synovial tissues has greater risk of developing secondary osteoporosis (OP). In particular, polymorphisms in *Adenosine Monophosphate Deaminase* 1 (*AMPD1*) and *Methylenetetrahydrofolate Reductase* (*MTHFR*) affect the outcome of methotrexate (MTX) treatment in patients with RA. Therefore, this study aimed to determine the association of *AMPD1* rs17602729, *MTHFR* C677T, and *MTHFR* A1298C polymorphisms with MTX activity in RA patients. A retrospective design was adopted to collect data from medical records and blood samples of 99 patients experiencing outpatient care at a referral hospital in Bandung. The inclusion criteria were patients diagnosed with RA, aged 18–59 years, and receiving MTX therapy for ≥ 6 months. DNA was isolated and then amplified using Polymerase Chain Reaction (PCR), and genotyping was performed with Sanger sequencing. The kinetic photometric method was used to measure the levels of calcium in the samples. The results showed that there is no significant association between the *MTHFR* C677T genotype variant or allele with calcium levels, as indicated by p-values of 0.177 and 0.174, respectively. The association between the *MTHFR* A1298C genotype variant or alleles with calcium levels was also not significant (p = 0.206 and p = 0.090, respectively). However, most patients had normal calcium levels (76 patients; 77.6%) with the *MTHFR* C677T genotype variant CC and the *MTHFR* A1298C genotype variant AA (84 patients; 84.9%). *AMPD1* rs17602729 in all patients had a CC genotype with normal calcium levels. The results suggested that there was no significant association between the genetic variation of *AMPD1* rs17602729, *MTHFR* C677T, and *MTHFR* A1298C with serum calcium levels in patients with RA receiving MTX therapy.

## Introduction

Rheumatoid Arthritis (RA) is a chronic and progressive autoimmune condition that affects synovial tissue attached to the skeletal tissue and joint cavities of the cartilage^1^. Its prevalence varies between 0.37 and 1.25% worldwide, and the highest is recorded in Northern European and North American countries, accounting for 29 cases/100,000 and 38/100,000, respectively. Indonesia has more than 1.3 million people affected by RA, with a total population of 268 million in 2020^2,3^. A common complication of arthritis similar to RA is osteoporosis (OP). The worldwide prevalence of secondary OP in RA patients reportedly ranges from 22 to 36%^4^. According to a previous study, this condition can be prevented by using effective medications and regular monitoring of bone minerals^5^. Calcium is a mineral that can be easily monitored, specifically in developing countries, such as Indonesia, and plays an important role in maintaining bone health^6^.

The treatments for RA are available in the form of conventional and biological disease-modifying antirheumatic drugs (DMARDs) to slow disease progression. Conventional DMARDs include methotrexate (MTX), leflunomide, sulfasalazine, and hydroxychloroquine. On the other hand, the biological DMARDs are divided into two groups, namely Tumor Necrosis Factor (TNF) inhibitor biologics, such as adalimumab, etanercept, and certolizumab, and non-TNF biologics, namely tocilizumab and rituximab^1,3^. According to a previous study, MTX is the most widely used first-line therapy and anchor of drugs for RA with a dose of 7.5–25 mg/week^3,7^.

MTX is a structural analog of folic acid that inhibits enzymes in the folate pathway, such as dihydrofolate and methylenetetrahydrofolate reductase (MTHFR). Dihydrofolate reductase reduces dihydrofolic acid to folinic acid, which is an MTX active intracellular metabolite. MTX inhibits aminoimidazole-4-carboxamide ribonucleotide (AICAR) transformylase, thereby increasing the AICAR levels. High AICAR levels lead to the release of adenosine, a potent anti-inflammatory mediator that suppresses inflammation in RA patients. MTX also modulates humoral and cellular immune responses in T and B cells, monocytes, neutrophils, as well as fibroblasts^8,9^.

Patients with RA are at a higher risk of OP due to several factors, such as age, post-menopause, glucocorticoid use, low body weight, calcium, and vitamin D, as well as immobility, and inflammatory reactions^10^. A previous study stated that genetic variation plays a significant role in the development of RA disease^11^. According to previous studies, certain genes and their polymorphisms, such as *Adenosine Monophosphate Deaminase* 1 (*AMPD*1) rs17602729, *MTHFR* C677T rs1801133, and *MTHFR* A1298C (rs1801131) may affect the occurrence of OP. However, *AMPD1* and *MTHFR* polymorphisms modulate the outcomes of MTX treatment^8,12–14^.

*AMPD1* (rs17602729), located on chromosome 1:114693436 (GRCh38.p14), converts *Adenosine Monophosphate* (AMP) to Inosine Monophosphate (IMP). However, in cases of deficiency, this conversion is inhibited, and adenosine is produced to mediate the anti-inflammatory effect of MTX. The production of adenosine is inhibited by MTX through the reduction of the AMPD enzyme^8,15,16^. The single nucleotide substitution in *AMPD1* rs17602729 was cytosine to thymine at the 34th nucleotide of exon 2 (C34T). These polymorphisms result in the termination of the glutamine codon substitution, thereby enhancing enzyme function. Consequently, patients with the CC homozygous genotype have higher skeletal muscle activity than CT and TT^12^.

MTHFR is a regulatory enzyme in the metabolic pathway of folic acid that degrades 5,10-methyltetrahydrofolate to 5-methyltetrahydrofolate. This enzyme is not directly inhibited by MTX but the intracellular distribution of folate and nucleic acid methylation can be disrupted by changes in its activity, which in turn affect effectiveness and toxicity^16–18^. *MTHFR* C667T and A1298C are the most common causes of toxicity in high-dose MTX therapy because they inhibit drug clearance^13^. The C667T polymorphism is caused by the substitution of alanine with valine. This substitution increases the intracellular concentration of homocysteine and changes the distribution of folic acid. Conversely, the A1298C polymorphism may cause a decrease in *MTHFR* gene expression due to the replacement of glutamic acid by alanine^14^.

Understanding the role of genetic variation as a risk factor for the outcome of MTX treatment is crucial in patients with RA. Calcium levels, as therapeutic outcome, were observed to determine the risk of secondary OP. This study is the first to identify the genetic profiles of *AMPD*1 (rs17602729) and *MTHFR* (C667T and A1298C) in patients with RA at a public hospital in Bandung, Indonesia.

## Results

### Patients characteristics

A total of 99 patients who met the inclusion criteria were included in the study, as shown in Table 1. Blood samples were collected from all the patients to test for serum calcium levels and gene polymorphisms. Among patients, approximately 79% had normal calcium levels in the range of 8.6–10 mg/dL, while the rest are outside the normal range (below or above).

**Table 1:** RA patients characteristics.

Characteristics	n (%)
Sex	
Male	10 (10.1%)
Female	89 (89.89%)
Age	
20–39	22 (22.22%)
40–59	77 (77.77%)
MTX dose	
7.5–15	63 (63.63%)
17.5–25	36 (39.39%)
Duration of therapy	
6–12 months	19 (19.19%)
13–24 months	18 (18.18%)
> 24 months	62 (62.62%)
Calcium level	
Normal	79 (79.79%)
Abnormal	20 (20.20%)

### DNA Visualization and Genotyping for AMPD1 rs17602729, MTHFR C677T and A1298C

**Figure 1:**
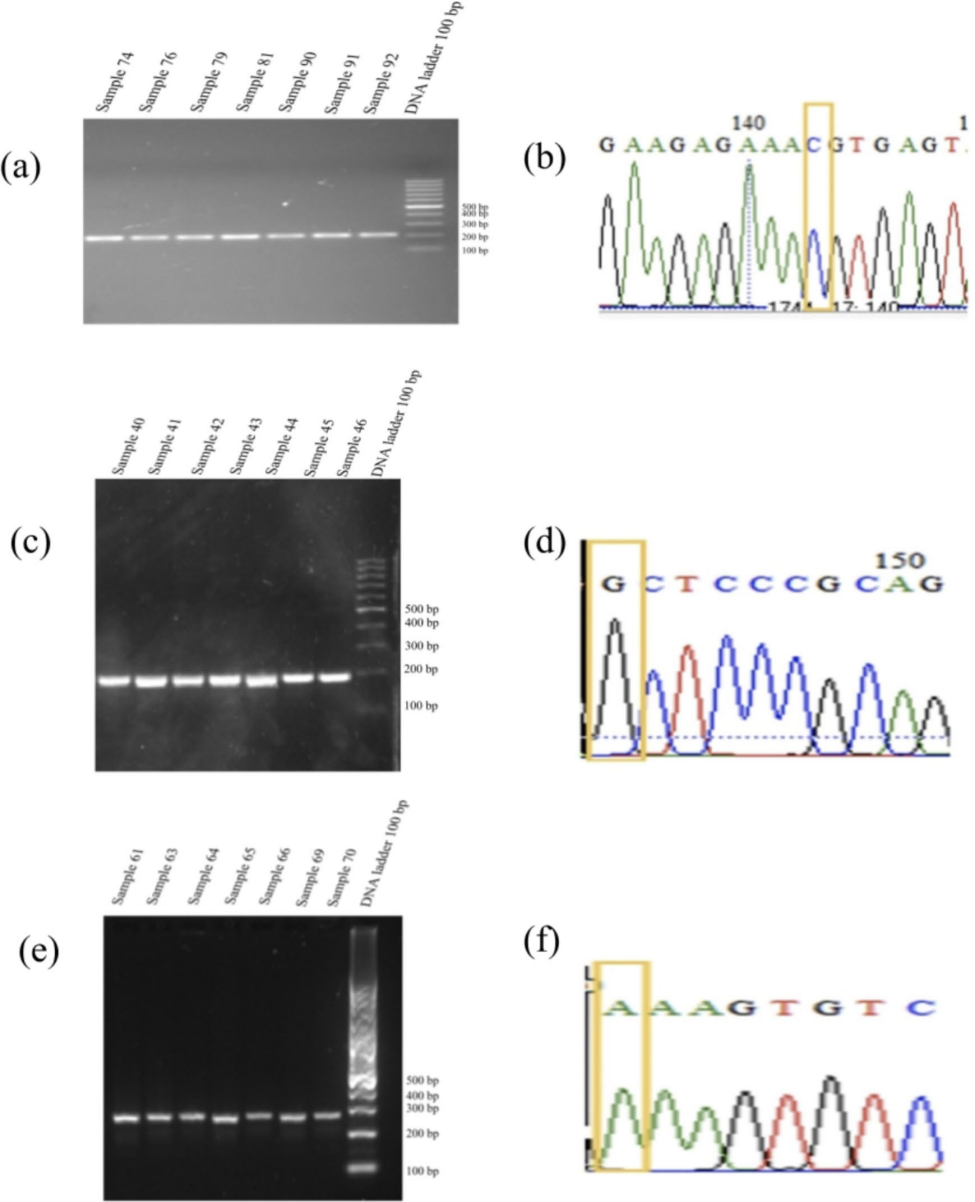
Visualization of PCR fragment using electrophoresis and confirmation by sequencing for (**a**) and (**b**) AMPD1 rs17602729, (c) and (d) MTHFR C677T, and (e) and (f) MTHFR A1298C.

PCR fragments were electrophoresed and visualized under UV light, as shown in Fig. 1 (refer to the Supplementary Info File Figure.pdf). The chromatogram of the sequencing was analyzed using the BioEdit application for the genotype of each sample. Patient data was obtained from the same place as the time of data collection and blood samples were carried out within a short period of time. The difference in numbers between the MTHFR C677T (n=98) and MTHFR A1298C (n=99) genes is because 1 patient's blood sample for MTHFR A677T could not show sequencing results even though several tests had been carried out. All sequencing sample results showed good chromatogram results, were characterized by low baseline noise and were distinct from the actual nucleotide peaks.

All samples tested were homozygous (CC) for *AMPD1* rs17602729, which precluded the calculation of Hardy–Weinberg equilibrium. In contrast, *MTHFR* C677T exhibited a wildtype genotype CC in 76 patients, CT in 18, and TT in 4, accounting for 77.6%, 18.4%, and 4.1%, respectively. Furthermore, *MTHFR* A1298C had 84.9% (n=84) genotype AA, 11.1% (n=11) AC, and 4% (n=4) CC. The genotype frequencies of *MTHFR* C677T and A1298C were in equilibrium, as showed by p-values of 0.045 and 0.0003 (p<0.05), according to the Hardy-Weinberg equation (Table 2).

**Table 2:** Hardy–Weinberg equilibrium for observed and expected genotype frequencies for MTHFR AC677T and A1298C.

MTHFR C677T			MTHFR A1298C		
Genotype	Observation	Expectation	Genotype	Observation	Expectation
CC	76 (77.6)	73.7244	AA	84 (84.9)	80.9116
CT	18(18.4)	22.5510	AC	11 (11.1)	17.1768
TT	4 (4.1)	1.7244	CC	4 (4)	0.91162
X^2^ value	3.9911		X^2^ value	12.8019122	
*P* value	0.045		*P* value	0.0003	

### Patients characteristic and frequency of MTX response (calcium serum levels) by AMPD1, MTHFR C677T, and A1298C genotypes

Statistical significance could not be calculated because all patients had the CC genotype of *AMPD1* rs17602729. As the result of MAF, AMPD1 rs17602729 gene cannot be calculated because the allele data obtained is homozygous (allele C). The MAF calculated for 12% of MTHFR C677T gene and 9% of MTHFR A1298C. Based on NCBI database SNPs reference, the allele frequencies of the polymorphisms in Asia population 99.95% for AMPD1 rs17602729, 66.14% for MTHFR C677T, and 75.3% for MTHFR A1298C. The association of *MTHFR* C677T with serum calcium levels as predictors of response to therapy showed p-values of 0.177 (genotype) and 0.174 (allele), respectively. *MTHFR* A1298C also had a p-value of 0.206 (genotype) and 0.09 (allele), as shown in Table 3. Normal serum calcium levels were found in 58 of 98 patients with the homozygous CC genotype. Meanwhile, MTX dose had a significant association (p = 0.022) with serum calcium values. Normal serum calcium levels were found in 64 of the 99 patients with the homozygous AA genotype. This study also considered other patient’s characteristics such as gender and age, however statistical analysis showed no significance association between age and gender to MTX outcome and gene polymorphisms (p>0.05). A total of 63 and 36 patients received a low and higher MTX dose of 7.5–15 mg/week and 17.5–22.5 mg/week, respectively. More than 50% had normal serum calcium values at low doses, as shown in Table 3. The association of genotypes on *MTHFR* A1298C and C677T with MTX dose did not have significant results (Table 4).

**Table 3:** Response of MTX (calcium serum levels) by AMPD1, MTHFR C677T, and MTHFR A1298C.

SNP	Calcium		*P* value
	Normal n/n total (%)	Abnormal n/n total (%)	
AMPD1 rs17602729			
Genotype			
CC	79/99 (79.8)	20/99 (20.2)	-
Allel			
C	158/198 (79.8)	40/198 (20.2)	-
MTHFR C677T			
Genotype			
CC	60/98 (78.9)	16/98 (21.1)	0.177
CT	14/98 (77.8)	4/98 (22.2)	
TT	4/98 (100)	0	
Allel			
C	134/196 (78.8)	36/196 (21.2)	0.174
T	22/196 (84.6)	4/196 (15.4)	
MTHFR A1298C			
Genotype			
AA	64/99 (76.2)	20/99 (23.8)	0.206
AC	11/99 (100)	0	
CC	4/99 (100)	0	
Allel			
A	139/198 (77.7)	40/198 (22.3)	0.090
C	19/198 (100)	0	
MTHFR A1298C & MTHFR C677T			
Genotype			
AA	63/83 (75.9)	20/83 (24.1)	0.797
CC	60/76 (78.9)	16/76 (21.1)	
AA + CC	46/62 (74.2)	16/62 (25.8)	
Allel			
A	137/177 (77.4)	40/177 (22.6)	0.849
C	134/170 (78.8)	36/170 (21.2)	
A + C	115/151 (76.1)	36/151 (23.8)	
Dosage of MTX (mg)			
7,5–15	51/99 (51.5)	12/99 (12.2)	0.022*
17,5–22,5	28/99 (28.3)	8/99 (8.1)

**Table 4:** Dose of Methotrexate and Polymorphism of MTHFR A1298C and C677T.

	MTHFR A1298C		MTHFR C677T	
	AA, n (%)	AC & CC, n (%)	CC, n (%)	CT & TT, n (%)
Low dose (n = 63)	52 (61.9)	11 (73.3)	50 (65.8)	13 (59.1)
High dose (n = 36)	32 (38.1)	4 (26.7)	26 (34.2)	9 (40.9)

There is no significance; p = 0.397 and p = 0.564.

## Discussion

The majority of the patients in this study had normal calcium levels, which served as an indicator of the response to MTX therapy and reduced the risk of secondary OP. RA treatment is aimed at slowing the process of bone erosion, repair, and rehabilitation. Corticosteroids, namely methylprednisolone 4 mg, are given for 3-6 months to all patients as bridging therapy and calcium supplements, some patients also receive vitamin D supplements so this can affect the patient's calcium levels. According to previous studies, bone erosion in RA can be repaired with conventional DMARDs, such as MTX^21,22^. This drug can slow down the development of bone erosion and possesses high efficacy and low toxicity^23–25^. The results of the study showed a significant relationship between serum calcium levels and MTX dose but not the duration of therapy. Conversely, the analysis of calcium values in association with *AMPD1* gene polymorphisms, *MTHFR* C677T and *MTHFR* A1298C, showed insignificant results. Patients with certain genetics/alleles showed a good therapeutic response to MTX.

Based on these results, all the patients had wild-type CC genotypes of *AMPD1* rs17602729. A total of 79 patients had normal serum calcium levels, while 20 had abnormal. A study in Poland found that the *AMPD1* rs17602729 CC genotype was the most common (CC, 53%, CT 43%, TT 4%) and fulfilled the Hardy–Weinberg equilibrium^12^. A Slovenian study also showed that the CC genotype was the most common and had a good response. However, patients with the T allele (CT and TT) showed a significant increase in DAS28 scores after 6 months of MTX monotherapy. A similar study conducted in Serbia showed that patients with the T allele of *AMPD1* rs17602729T had lower DAS28 values. Several studies have also found no significant response to therapy. The relationship between genes and response to therapy is affected by several factors, such as the dose of MTX or the condition of the patient at diagnosis^26–28^.

The *MTHFR* C677T and A1298C polymorphisms are associated with decreased enzyme activity^29^. In this study, most of the genotype frequencies obtained were wild-type 1298AA and 677CC, with normal serum calcium levels, suggesting a good response to prevent bone erosion. Similar genotype frequencies have also been reported by other studies, such as the AA of *MTHFR* A1298C and the CC of *MTHFR* C677T, which were the most common genotypes. The results fulfilled the Hardy-Weinberg equilibrium^30^.

In a study conducted in Japan, the majority of outpatients who received low doses of MTX and were assessed after six months had the CC genotype and C allele. Those with the 677C allele responded well to therapy^31^. Another study in India showed that patients with the CC or C alleles responded well^32,33^. Several other studies have also reported no association between *MTHFR* 677CC and efficacy. In this study, patients with the CC genotype have normal calcium levels. Furthermore, serum calcium levels were inversely correlated with bone mineral density (BMD). This shows that when the BMD value was low, calcium levels increased^34^.

Similar to *MTHFR* C677T, several studies have reported different results with *MTHFR* A1298C. In this study, most patients had the AA genotype with a good response and normal serum calcium levels but was not significant. A study conducted for two years in the Netherlands also showed that the AA genotype had a good response and clinical improvement, while patients with the C allele had a risk of MTX side effects^35^. This result is consistent with the report of a study by Kato et al. in Japanese patients, which showed a good response to the AA genotype^36^. In contrast, two previous studies in Japan reported that the C allele and the CC genotype responded well to low doses of MTX^31,37^.

A report in Mexico showed no significant association between *MTHFR* A1298C and OP incidence in RA patients^38^. In Algeria, the frequency of the *MTHFR* A1298C genotype was not significantly different but the A allele exhibited a better response. Allele A showed a good/moderate EULAR response to MTX, while C was significantly poor^39^.

*MTHFR* A1298C and CC genotypes on *MTHFR* C677T were also investigated in this study. A total of 62 patients had both, while 46 had normal calcium levels. Furthermore, 151 patients had both the A and C alleles of these genes, with 115 having normal calcium values. The results showed that there was no significant difference between patients who had one genotype or allele and those with both.

The dose relationship was found to have significance on calcium values. The dose of MTX that is effective in suppressing RA activity can vary greatly between patients but MTX treatment is almost ineffective in some cases. RA patients were grouped based on the MTX dose used and the relationship with gene polymorphisms was analyzed. According to previous studies, *MTHFR* is a gene that may affects the response to treatment with MTX^13,31,37^.

A study by Urano in Japan showed that some patients received high doses of MTX because of a poor response in controlling RA, while others had good responses only with low doses. Therefore, patients were grouped based on their genotype and analyzed for their relationship with dose. The results showed that RA patients with the C allele (CC & CT) in *MTHFR* A1298C had more good response to therapy, necessitating lower MTX doses compared to patients lacking the C allele (AA). However, *MTHFR* C677T did not show a significant difference in allele frequency^31^.

Another study in Japan by Taniguchi showed that patients with the C allele in *MTHFR* A1298C (AC & CC) had a better response to therapy. These data showed the influence of gene dosage on enzyme activity and folic acid concentration. The effect of gene dosage is important in individualized medicine based on pharmacogenomics by classifying patients^37^.

This study assessed the response of MTX therapy in reducing the risk of secondary OP through calcium values due to the limitations of other tests. Further investigations are recommended to use larger sample sizes, considering the various ethnic groups spread across islands in Indonesia. Supportive data, such as BMD, DAS28, RANKL, and OPG values, are needed. In addition, the influence of *MTHFR* genotype on the effectiveness of MTX must be supported by pharmacokinetic values. Despite these limitations, this is the first study in Indonesia to observe a correlation between genetic variation (*AMPD1* and *MTHFR*) in patients with RA.

## Conclusion

In conclusion, all patients in this study exhibited the CC genotype for *AMPD1* rs17602729, while the majority had CC and AA for *MTHFR* C677T and A1298C, respectively. The results suggested that there was no significant association between the genetic variation of *AMPD1* rs17602729, *MTHFR* C677T, and *MTHFR* A1298C and serum calcium levels in patients with RA receiving MTX therapy.

## Methods

### Study design

This observational retrospective study was conducted by collecting data from the medical records of the patients at the largest referral government hospital in West Java. This province is the most populous in Indonesia and covers approximately 48,220,094 million people^19^. The study group consisted of 99 outpatients from the rheumatology clinic of the Hasan Sadikin Hospital, Bandung. The patients were diagnosed with RA by a rheumatology specialist using DAS28, same race, aged 18–59 years, receiving MTX therapy for ≥6 months, and registered in the rheumatology clinic from 2011 to 2021. The data were excluded for patients with comorbidities or experiencing treatments susceptible to AMPD1 and MTHFR polymorphisms, as well as variations in calcium levels. Secondary data were obtained from medical records and consisted of patients demographic data (age and sex). It also contains laboratory examination results comprising calcium serum levels by the kinetic photometric method, patients medication (types of medicines, dosage, and duration of therapy), and medical history (comorbidities)**.**

This study was conducted in accordance with the Declaration of Helsinki and ethical approval was obtained from the Research Ethics Committee of Universitas Padjadjaran (approval number: 423/UN6. KEP/EC/2020). Written informed consent was administered and obtained from all patients who participated. Patient data were analyzed anonymously, and no personal data were collected.

### DNA isolation and genotyping of AMPD1 (rs17602729) and MTHFR (C667T and A1298C)

DNA was isolated using *GenEluteTM Mammalian Genomic DNA Miniprep Kit Protocol *G1N70 (Sigma-Aldrich, St.Louis, USA ) and the extracted samples were stored at 20 °C until further use. DNA was tested quantitatively using a spectrophotometer *Infinite M200 Pro* (Tecan, Grödig, Austria) at an absorbance of 260 nm and a ratio of 260/280 nm to determine quantity and purity.

The extracted DNA was amplified by Polymerase Chain Reaction (PCR) using specific primers for each gene. The primers used for *AMPD1* (rs17602729) were forward primer 5′-CTCTGACAAATGGCAGCAAA-3′ and reverse primer 5′-ATTCCCAAGCTTTCTGATGG-3′^20^. Meanwhile, *MTHFR* C667T used forward primer 5’-TGAAGGAGAAGGTGTCTGCGGGA – ‘3, and reverse primer 5’- AGGACGGTGCGGTGAGAGTG – ‘3. For *MTHFR* A1298C, the forward and reverse primer were 5’- CATTCCGGTTTTGGTTTCCCC– ‘3 and 5’- AGTCAGGGGCAATTTACAGG– ‘3, respectively. The gene sequences were obtained from the National Center for Biotechnology Information (NCBI) website (http://www.ncbi.nlm.nih.gov) and inputted into the Primer Designing Tool at www.ncbi.nlm.nih.gov/tools/primer-blast/. Specific primers were confirmed using the Oligo Calc Oligonucleotide Properties Calculator (http://www.basic.north western.edu/biotools/oligocalc.html). PCR was carried out using PCR Master Mix GoTaq® Green Master Mix (Promega, Madison, USA), and a 2 µL DNA template. The annealing temperature for *AMPD1* (rs17602729), MTHFR C667T, and MTHFR A1298C were 56 $^\circ{\rm C}$, 61.3 $^\circ{\rm C}$, and 61 $^\circ{\rm C}$, respectively. The products were analyzed qualitatively using 2% agarose gel containing DNA Staining (SYBRTM Safe DNA Gel Stain, Thermo Fisher, Waltham, USA), at 80 V for 90 min, as well as 100 bp DNA ladder. Subsequently, the products were observed under UV light at a wavelength of 312 nm and sequenced.

### Statistical analysis

Genotyping distribution data were analyzed using the Hardy-Weinberg equilibrium equation (HWE, df = 1). Kruskal-Wallis test was used to assess the relationship between *MTHFR* A1298C and *MTHFR* C677T gene polymorphisms and calcium levels. Meanwhile, *AMPD1* was not tested for statistical analysis because all patients had the same genotype.

### Ethics approval and consent to participate

This study was conducted in accordance with the Declaration of Helsinki and ethical approval was obtained from the Research Ethics Committee of Universitas Padjadjaran (approval number: 423/UN6. KEP/EC/2020). Written informed consent was obtained from all patients who participated.

## Supplementary Information

Supplementary Figures.

## Data Availability

The data that support the results of this study are available from the corresponding author upon reasonable request.
