# Supplementary material for: AMPD1 and MTHFR genes are not associated with calcium levels in rheumatoid arthritis patients with methotrexate therapy in Indonesia
Source: Sci Rep. 2025 Jan 10;15:1647. doi: 10.1038/s41598-024-69604-z (PMC11723994; doi:10.1038/s41598-024-69604-z)
Supplement: Supplementary file 1 — Supplementary Figures. [file 41598_2024_69604_MOESM1_ESM.pdf]

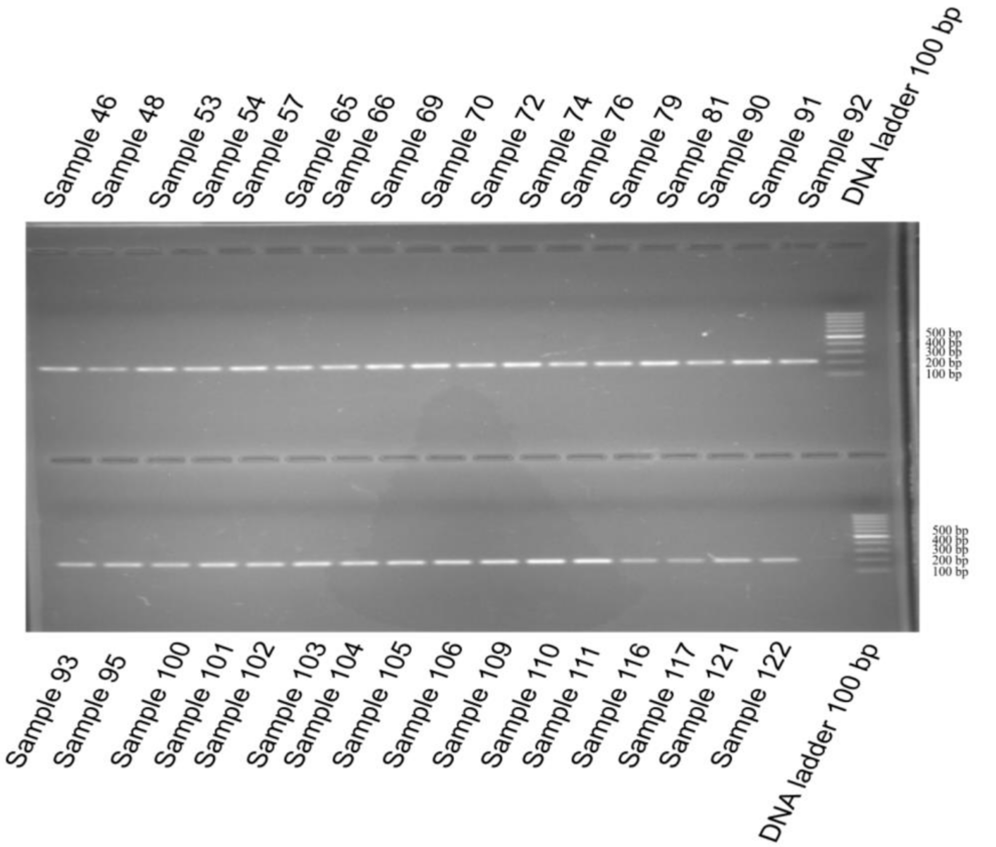

Figure 1. Visualization of PCR fragment using electrophoresis and confirmation by sequencing for AMPD1 rs17602729 (Size marker210 bp)

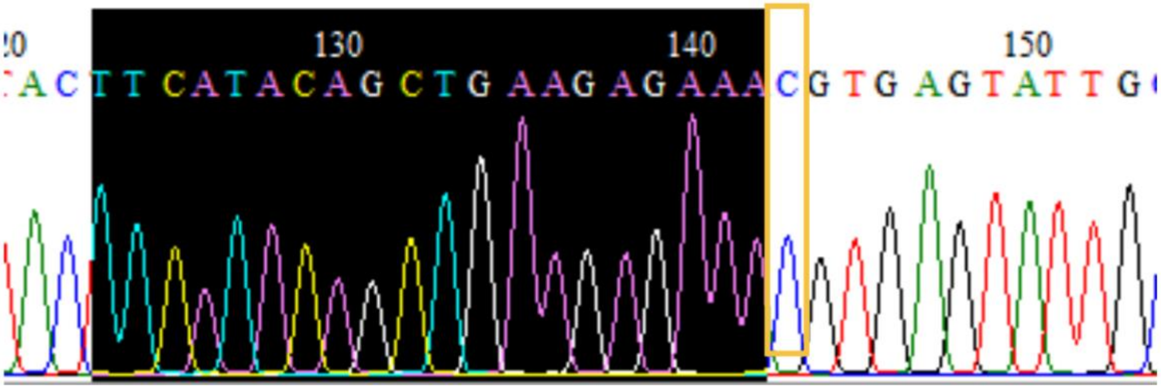

Figure 2. Visualization of PCR fragment using electrophoresis and confirmation by sequencing for AMPD1 rs17602729

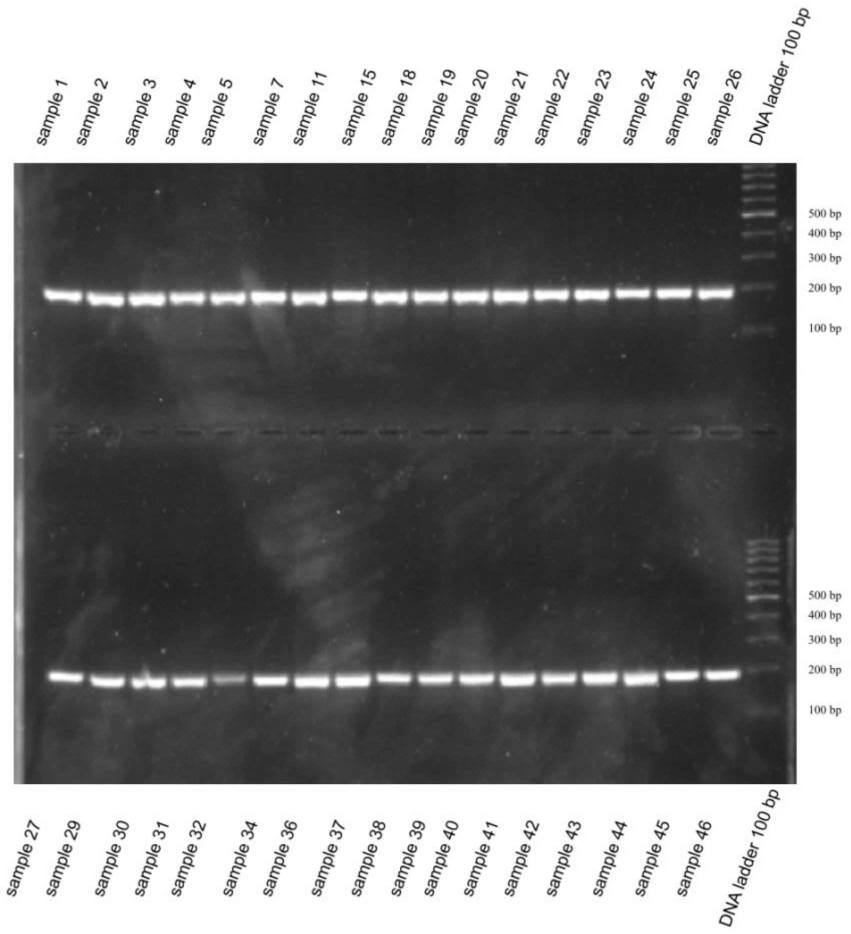

Figure 3. Visualization of PCR fragment using electrophoresis and confirmation by sequencing for MTHFR C677T (Size marker 198 bp)

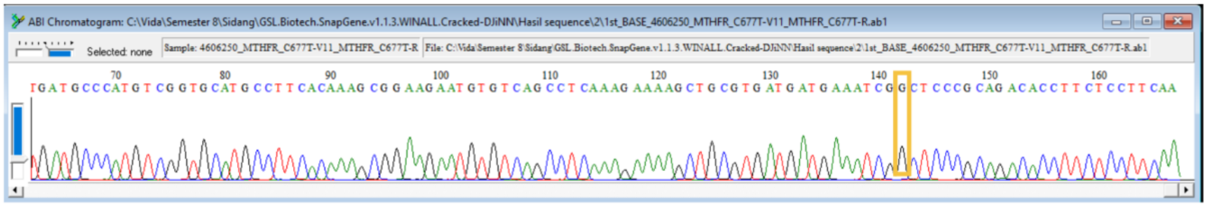

Figure 4. Visualization of PCR fragment using electrophoresis and confirmation by sequencing for MTHFR C677T

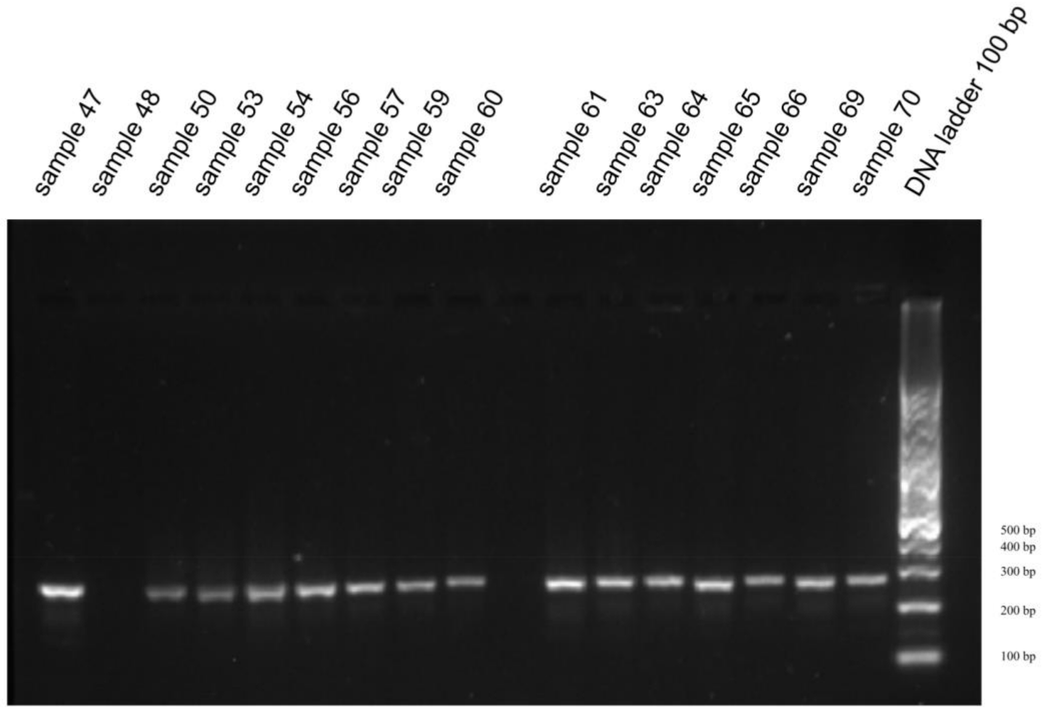

Figure 5. Visualization of PCR fragment using electrophoresis and confirmation by sequencing for MTHFR A1298C (Size marker 270 bp)

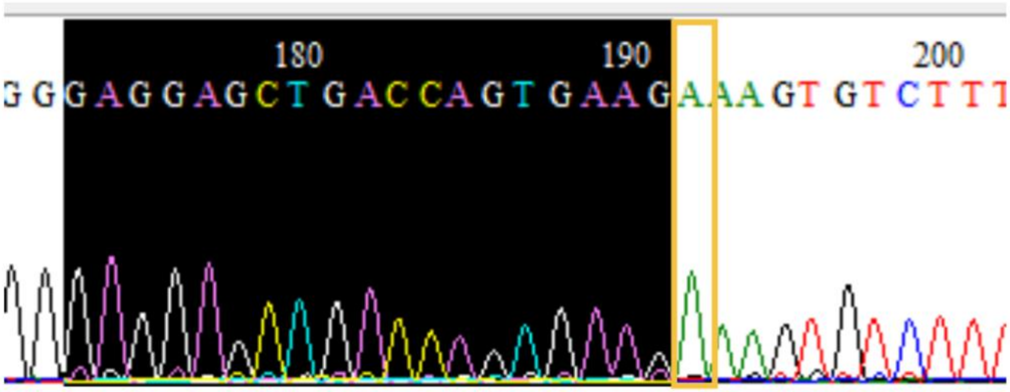

Figure 6. Visualization of PCR fragment using electrophoresis and confirmation by sequencing for MTHFR A1298C
